# Long-Acting Injectable Second-Generation Antipsychotics Improve Negative Symptoms and Suicidal Ideation in Recent Diagnosed Schizophrenia Patients: A 1-Year Follow-up Pilot Study

**DOI:** 10.1155/2018/4834135

**Published:** 2018-08-30

**Authors:** Valentina Corigliano, Anna Comparelli, Iginia Mancinelli, Benedetta Montalbani, Dorian A. Lamis, Antonella De Carolis, Denise Erbuto, Paolo Girardi, Maurizio Pompili

**Affiliations:** ^1^Department of Neurosciences, Unit of Psychiatry, Sapienza University of Rome, School of Medicine and Psychology, Via di Grottarossa 1035-39, 00198 Rome, Italy; ^2^Department of Psychiatry and Behavioral Sciences, Emory School of Medicine, Grady Hospital, Atlanta, GA, USA; ^3^Department of Neurosciences, Unit of Neurology, Sapienza University of Rome, School of Medicine and Psychology, Via di Grottarossa 1035-39, 00198 Rome, Italy

## Abstract

Long-acting injectable second-generation antipsychotics (LAI-SGA) are typically used to maintain treatment adherence in patients with chronic schizophrenia. Recent research suggests that they may also provide an effective treatment strategy for patients with early-phase disease. The aim of this study is to evaluate clinical and psychosocial outcomes among recent and long-term diagnosed schizophrenia outpatients treated with LAI-SGA during a follow-up period of 12 months. Stable schizophrenia patients receiving LAI-SGA with 5 or less years of illness duration (*n *= 10) were compared to those with more than 5 years of illness duration (*n *= 15). Clinical data was assessed through the Positive and Negative Syndrome Scale (PANSS), the Global Assessment of Functioning (GAF), the Columbia Suicide Severity Rating Scale (C-SSRS), the Recovery Style Questionnaire (RSQ), and the Mayer-Salovey-Caruso Emotional Intelligence Test (MSCEIT) Managing Emotion branch. Recently diagnosed patients showed greater improvement versus patients diagnosed for more than 5 years in adjusted mean GAF score, in PANSS factor score for negative and depressive symptoms, and in severity and intensity of suicidal ideation. Our preliminary findings support the hypothesis that LAI-SGA may influence the course of the illness if administered at the early phase of the illness. However, replicate studies are needed, possibly with larger samples.

## 1. Introduction

Schizophrenia poses a significant burden to the patient, caregiver, and society in general. Additionally, mostly due to suicide deaths, patients diagnosed with schizophrenia have their life expectancy reduced by approximately 10 years [1].

Much of the deterioration in schizophrenia occurs within the first 5 years of disease onset [2], suggesting that the early stages are a critical period for effective treatment.

Treatment for schizophrenia aims to reduce the severity of symptoms, prevent the recurrence of episodes, and provide support to allow for an appropriate level of functioning. Given that up to half of patients suffering from schizophrenia may not take their medications as prescribed, treatment adherence is a major challenge [3], with serious consequences on the course of the illness [4–6]. Nonadherence is of particular significance to patients in the early phases of psychosis. As the disease progresses, deterioration in treatment responsiveness becomes more common, especially with standard oral antipsychotics. Long-acting injectable (LAI) second-generation antipsychotics were developed with the primary objective of addressing both hidden and overt nonadherence. Although LAI-SGA traditionally have been reserved for patients at later periods of their disease, increasingly, LAI-SGA are being suggested for early episodes of schizophrenia to reduce the risk of relapse and long-term disability associated with the chronic course of the illness [7–9]. Furthermore, available treatments are effective in reducing positive and affective symptoms; however, negative and cognitive symptoms, which affect global functioning and outcome, still represent an unmet need. Despite the importance of effective treating of negative symptoms, few studies have explored the changes in negative symptomatology among nonacute schizophrenia patients who switched to LAI-SGA from oral antipsychotics [10]. Thus, more research is needed to investigate this clinical outcome.

A recent review indicated a possible association between treatment with LAI-SGA and reduced suicide risk in schizophrenia [11], with LAI-SGA improving various risk factors for suicide. However, although there is a well-documented association between schizophrenia and suicide risk factors, there is a lack of studies investigating the efficacy as well as the effectiveness of LAI-SGA in suicide risk among schizophrenia patients.

Some researchers have proposed that social cognition deficits may be a schizophrenia vulnerability marker [12] affecting real-life functioning in people [13]. To date, no differences have been found in social emotion cognition task performance after treatment with LAI-SGAs in patients with schizophrenia [14]. However, the lack of empirical data on this relationship did not allow for definitive conclusions to be made.

In the treatment of patients with schizophrenia, the primary goal is traditionally clinical recovery, which includes remission of symptoms and functional improvement. The term personal recovery is used to describe the patient-based definition of recovery [15]. The construct of personal recovery has been developed based on narratives of individuals who have experienced mental illness [16]. To our knowledge, no studies have explored the effect of antipsychotic drugs on personal recovery.

The main aim of the present study is the evaluation of negative symptoms, social cognition, and global functioning 12 months after the initiation of treatment with LAI-SGA among patients who had a recent diagnosis of schizophrenia compared to long-term schizophrenia patients. As secondary outcome, we aim to compare changes in suicide risk and personal recovery between the two groups to determine LAI-SGA effectiveness on subjective well-being. We hypothesized that recently diagnosed patients would improve more than long-term diagnosed schizophrenia patients on psychiatric and psychosocial features affecting illness prognosis.

## 2. Materials and Methods

### 2.1. Subjects

We enrolled patients meeting the DSM-IV schizophrenia disorder based on the Structured Interview for DSM-IV Disorders-I (SCID-I) [17] who were administered long-acting antipsychotic treatment based on an attending physician's clinical judgment. Patients were recruited from the outpatient service of the Psychiatric Department of the Sant'Andrea Hospital, Rome. Patients were required to be clinically stable for a minimum of 4 months and receiving consistent doses of oral antipsychotic medication (excluding clozapine) for 4 weeks prior to study entry. Patients were allowed to continue on any prescribed antidepressants, anxiolytics, or mood stabilizers during the trial, provided they had been on a stable dose for 4 weeks before baseline.

Of the 35 eligible patients, 7 dropped out before completing the procedures and 3 were excluded from the follow-up because of the pharmacological treatment change over the observational period of the study, which left 25 to be included in the analyses (19 males, 6 female). Of the final sample of patients, 14 received treatment with paliperidone palmitate (PLAI), 2 with risperidone long-acting (RLAI), 2 with olanzapine pamoate (OLAI), and 7 with aripiprazole long-acting (ALAI). Six patients used cannabis (24%) and 9 reported suicidal ideation at the time of the assessment, two of which made a suicide attempt during the course of the illness. There was no change in the antipsychotic medication dosage during the course of the study. Two patients were hospitalized because of psychotic relapse, but they were not excluded because there was no change in the LAI treatment. Patients were categorized by the years of illness duration: those with 5 or less years of illness were classified as recent diagnosis schizophrenia (recent SZ) and those with more than 5 years of illness were classified as long-term diagnosis schizophrenia (long-term SZ). The total numbers of patients with at least 12-month follow-up in the recent diagnosis SZ and long-term diagnosis SZ groups were 10 and 15, respectively.

Patients were excluded based on the following criteria: (1) current or past comorbid diagnosis of autistic disorder or other pervasive developmental disorder, (2) history of severe head injury, (3) severe medical conditions or major neurological disorders, including mental retardation and dementia, and (4) any current drug abuse with the exception of cannabis, because of the high prevalence of its use among schizophrenia patients [18, 19]. Patients were tested at baseline and prospectively followed up to 12 months. Written informed consent was obtained from all participants after providing a complete description and explanation of the study. The study received approval from the hospital's ethical committee.

### 2.2. Assessments

Data on sociodemographic and psychopathological variables were collected at clinical interview. Psychopathology was assessed with the PANSS [20]. As recommended by Wallwork et al. [21], we used the PANSS to extract the following 5 factors: (a) positive (POS) (P1, delusions; P3, hallucinatory behavior; P5, grandiosity; and G9, unusual thought content); (b) negative (NEG) (N1, blunted affect; N2, emotional withdrawal; N3, poor rapport; N4, passive withdrawal; N6, lack of spontaneity; and G7, motor retardation); (c) disorganized/concrete (DIS) (N5, difficulty in abstract thinking; P2, conceptual disorganization; and G11, poor attention); (d) excited (EXC) (P4, excitement; P7, hostility; G8, uncooperativeness; and G14, poor impulse control); and (e) depressed (DEP) (G2, anxiety; G3, guilt feelings; and G6, depression).

Global functioning was assessed with the Global Assessment of Functioning (GAF) Scale [22]. The GAF assesses the psychological, social, and occupational functioning of a patient on a 100-point scale ranging from 1 (severe functional impairment) to 100 (optimal functioning), with higher scores indicating better functioning.

Suicide risk was assessed with the Columbia Suicide Severity Rating Scale (C-SSRS) [23] “Since Last Visit” version. The C-SSRS has four constructs relevant to recent suicidal ideation (SI): (1) severity (1=wish to be dead, 2=nonspecific active suicidal thoughts, 3=suicidal thoughts with methods, 4=suicidal intent, and 5=suicidal intent with plan); (2) intensity (sum across six items each rated 0 to 5: most severe ideation, frequency, duration, controllability, deterrents, and reason); (3) behavior; and (4) lethality.

Personal recovery was measured with the Recovery Style Questionnaire (RSQ) [24], a 39-item self-report measure, designed to reflect categories consistent with those developed by McGlashan et al. [25]. Thirteen scales were computed, with higher scores representing “integration” (i.e., a recovery style associated with better outcome, less depression, and better self-evaluation, as compared to a “sealing-over” style) [26]. Different from personal recovery, clinical recovery is related to improvement of symptoms and functioning.

Social cognition was assessed with the Mayer-Salovey-Caruso Emotional Intelligence Test (MSCEIT) Managing Emotion branch [27], which includes subscales rating which emotional strategy would be most effective for regulating the self and other people's emotions. For each item, participants responded on the level of effectiveness of a list of options, ranging from 1 (very ineffective) to 5 (very effective), or the presence of a certain emotion, ranging from 1 (not at all present) to 5 (present to a great extent).

### 2.3. Statistical Analysis

We examined differences in demographic characteristics and clinical features at baseline between the recent SZ and the long-term SZ patients. Then, we conducted Student's* t*-tests to assess changes in psychopathological, functional, and personal resources and context-related factors from baseline (T0) to follow-up (T1) in each group. An analysis of covariance model with group as a factor and baseline scores as a covariate was used to assess between-group changes from baseline to end point with no correction for multiplicity. Mean changes from baseline were reported as least squares means, adjusted for group and baseline values. A significance level of 0.05 was used for all statistical tests and two-tailed tests were applied. All analyses were conducted with the statistical package SPSS (version 17.0.2).

## 3. Results

The mean age was significantly lower in the recent SZ group compared to the long-term SZ group. No other differences in demographic variables were found between groups. The clinical variables in both groups were similar at baseline and no significant differences were found (Table 1). Considering pharmacological treatment, according to Gardner et al. [28], mean daily dose for antipsychotic medication in chlorpromazine-equivalents was 19.3 mg (DS=3.9) in recent SZ and 22.9 mg (DS=4.9) in long-term SZ, with no significant difference between the two dosages (T 2.41, df=23, p>0.05). Thirty percent of recent SZ patients and 33% of long-term SZ patients received mood stabilizers at baseline, whereas 10% of recent SZ and 20% of long-term SZ received anxiolytic treatment at the same time. No patients in both groups received antidepressant at the baseline and during the course of the study.

In the recent SZ group, a significant improvement was found on the negative PANSS dimension, severity, and intensity of SI and on the RSQ (see Table 2). Both groups had a significant improvement on GAF score and PANSS factor score for positive and excitement symptoms from baseline to 12-month follow-up. There were no differences on MSCEIT scores from baseline to 12-month follow-up in either patient group. Mean changes in evaluated factors for both groups are reported in Table 2.

ANCOVA analyses showed greater improvement in GAF and in PANSS negative and depressive factor scores in the recent SZ group versus the long-term SZ group. The recent SZ group had lower severity and intensity SI after SGAI-LAI treatment compared to the long-term SZ group. No other statistically significant differences were found among groups (see Table 3).

## 4. Discussion

The aim of this pilot study was to compare clinical, cognitive, and psychosocial outcomes between recent and long-term diagnosed schizophrenia patients after 12 months with second-generation long-acting antipsychotic drugs. On the whole, our findings show the efficacy of LAI-SGA in improving the positive and excited symptoms and general functioning at 12 months of follow-up.

This is in line with most of the literature demonstrating the effectiveness of LAI-SGA in schizophrenia patients regardless of the stage of the illness [29–31].

Interestingly, in our study, schizophrenia patients at early phase of the illness seemed to benefit more from the use of LAI-SGA compared to chronic patients. In fact, only the group of recent SZ patients reported an improvement of negative symptoms, a reduction in intensity and severity of suicidal ideation, and a more effective recovery style. Furthermore, greatest improvements were found among those patients who started LAI-SGA within 5 years of diagnosis in PANSS negative and depressive factors, in global functioning, and in severity and intensity of SI. Benefits of early treatment have already been shown with oral antipsychotics [32, 33], whereas there are fewer studies examining the long-term benefits of LAI in recently diagnosed schizophrenia. Existing studies have demonstrated that recently diagnosed patients treated with LAI-SGA had a global higher treatment response and improved functioning as compared to chronic patients at six months of follow-up [10, 34, 35]. Unfortunately there are no studies examining the outcome of specific symptom domains through the analysis of single PANSS factors, nor the suicidal ideation or other clinical variables that influence the outcome of the disease. Moreover, in the majority of studies, follow-up is limited to six months, which is a relatively short outcome making the comparison weaker. Nonetheless, our findings are in line with other studies that show a global improvement in the recent diagnosed schizophrenia group.

Improvement of negative symptoms in recently diagnosed patients is the most important finding of our study. Negative symptoms have long been conceptualized as a core aspect of schizophrenia, playing a key role in the functional outcome of the disorder and representing a significant unmet need, mostly if persistent over the first episode of psychosis [36].

There are several indications that second-generation antipsychotics have promyelinating [37] and lipogenic effects [38] of importance to the therapeutic response in psychotic disorders. A recent study found a significant relationship between the increase in HDL and decrease in negative symptoms in FEP patients after one year of antipsychotic medication, independently of weight change [39]. It has been speculated that HDL in serum could be considered an indirect proxy for cholesterol in the brain, where improved supply could play a positive role in myelination. Patients with schizophrenia have reduced myelination in the brain [40–42], and in vivo MRI studies have demonstrated in FEP promyelinating effects of second-generation antipsychotic drugs specifically in their long-acting formulation [37, 43, 44] that in part could be related to their lipid-stimulating effects [39, 45].

The second noteworthy finding of the present study is the reduction of severity and intensity of SI only in the early phase of the disease. This finding supports the proposals advanced in a recent review [11] that LAI treatments may potentially be an important strategy in suicide prevention by indirectly acting on a range of suicide risk factors in schizophrenia.

Suicide-related mortality is higher among subjects recently diagnosed with schizophrenia (≤5 years from diagnosis) [46]. Symptoms of depression are consistently supported by factors involved in suicidal ideation [47, 48]. Even if suicidal ideation may be present in different stages of disease, some differences have been described between the risk of suicide in patients experiencing first episode of psychosis and those with long-term schizophrenia. Over the course of the illness, suicide risk is related to the loss of social role and functioning mostly due to neurocognitive impairment. On the other hand, in the early phase of the illness, suicidal ideation may be related to the impact of first symptom experiences, to the valence of personal meaning-making, and to the consequences of the diagnosis of psychosis, all being issues concerning the subjective experience of the illness.

Beyond the reduction of depressive symptoms, the reduction of suicidal ideation may be the result of the improvement of personal recovery style. In fact, whereas clinical recovery improved in both groups, personal recovery improved only in the recent SZ group. In schizophrenia, personal recovery interferes with both symptom reduction and social functioning [49] and it has been recently found that suicide ideation is less prevalent among individuals with schizophrenia that self-reported greater recovery [50]. McGlashan [51] conceptualized the subjective experience of psychosis as a continuum of recovery styles. Two distinct recovery styles (i.e., “integration” and “sealing over”) have been defined [24, 25, 51]. Patients who employ the “sealing-over” recovery style make more negative self-evaluations and perceive their parents as significantly less caring than those with the “integration” style [52]. This latter style seems to favor recovery [51]. At one end of the continuum lies “integration,” which is exemplified by persons who show an interest in their psychotic experiences and appear eager to discuss and learn more about them and to gain a meaningful perspective on them. At the other end of the continuum is “sealing over,” exemplified by persons who have difficulty recalling or describing the phase of acute psychosis, deny the existence and/or severity of their illnesses, and expect to return rapidly to normal functioning. From the subjective perspective, recovery is driven by individual's lives, peer support, and subjective experiences of mental illness and recovery and entails much more than managing symptoms. Precisely for that reason, when recovery style tends toward integration, it may positively affect suicidal ideation. Thus, we suggest that outcome measures in clinical practice, which currently focus on symptom remission and functioning, should be extended to include personal recovery. Several limitations have to be taken into account. First, the small sample size increased the risk of biased findings. However, given the pilot nature of the present study, the results obtained in this investigation should be regarded as preliminary. Second, as the study was not randomized, a selection bias cannot be ruled out. Nevertheless, the selection of a real-life, noninterventional study design allows obtaining data applicable to daily clinical practice. Lastly, the definitions of “recent” versus “long-term” schizophrenia are arbitrary time points that may not take into account potential differences in treatment duration and history prior to the start of LAI-SGA. Indeed, much of the deterioration in schizophrenia occurs within the first 5 years of disease onset [32], suggesting that the first stage within 5 years is a critical period for effective treatment.

The major strength of this study is the use of the PANSS five-factor consensus model as outcome parameter, instead of considering clinical global measures. Other studies have focused on first-episode schizophrenia or have been limited to 6-month duration [53–55], whereas this study has a longer follow-up time.

To our knowledge, this pilot study is the first one providing initial evidence that LAI-SGA specifically improves negative symptoms, suicidal ideation, and personal recovery in the early phase of the illness and supports the hypothesis that LAI-SGA may potentially modify the course of the illness if administered at first episode. Studies on the pathophysiology of schizophrenia suggest that clinical deterioration and “deficit processes” may be irreversible if left untreated, resulting in further functional decline and neurological impairment. Replicability of our findings with larger samples is a crucial next step in order to accurately determine the potential benefit of LAI-SGA to address negative symptoms and suicidal ideation in the early phase of schizophrenia.

## 5. Conclusions

In this pilot study we found that, in recent diagnosed schizophrenia negative symptoms, suicidal ideation and personal recovery improved with LAI-SGA treatment, with more improvements on global functioning and negative and depressive symptoms compared to long-term diagnosed schizophrenia, supporting the hypothesis that LAI-SGA may influence the course of the illness if administered at first episode. However, replicate studies are needed, possibly with larger samples.

## Figures and Tables

**Table 1:** Demographics and baseline diseasecharacteristics of Recent and Long-term diagnosed SZ group.

	**Recent SZ (n=10)**	**Long-term SZ (n=15)**	**Analyses**		
	Mean (SD)	Mean T0 (SD)	F	df	p
**Age**	25.7 (6.5)	43.3 (10.6)	3.618	23	0.001
**Years of education**	11.9 (2.1)	12.9 (2.4)	0.130	23	0.316
**Time since diagnoses (years)**	3.5 (1.3)	17.73 (9.4)	9.636	23	0.002
**GAF **	43.6 (7.8)	40.1 (11.3)	0.797	23	0.408
**PANSS Pos**	16.2 (6.8)	20.7 (6.9)	0.035	23	0.117
**PANSS Neg**	17.5 (7.0)	21.1 (6.9)	0.045	23	0.834
**PANSS Gen**	36.5 (7.1)	36.7 (11.2)	1.466	23	0.238
**PANSS Tot **	70.2 (11.4)	78.5 (23.6)	3.985	23	0.058
**IS severity**	1.9 (2.3)	1.1 (2.1)	1.007	23	0.411
**IS intensity**	6.8 (8.4)	3.9 (7.1)	1.210	23	0.378
**RSQ**	21.2 (6.1)	22.4 (3.9)	1.288	23	0.553
**Social cognition**	80.6 (7.9)	81.9 (12.1)	4.383	23	0.756

**Table 2:** Mean changes in clinical and psychosocial features from baseline to 12-month follow-up in recent schizophrenia and in long-term schizophrenia patients.

	**Recent SZ (n=10)**	**Long-term SZ (n=15)**
	Mean T0 (SD)/Mean T1 (SD)	Mean T0 (SD)/Mean T1 (SD)
**GAF **	**43.6 (7.8)/56.4 (9.5)** **∗**	**40.1 (11.3)/49.0 (7.5)** **∗** **∗**

**PANSS Pos**	**9.2 (4.6)/6.8 (3.8)** **∗**	**11.5 (4.5)/6.6 (2.5)** **∗** **∗**

**PANSS Neg**	**13.7 (6.7)/11.5 (5.1)** **∗**	16.7 (6.6)/15.1(5.0)

**PANSS Dis/Con **	7.0 (1.8)/5.8 (1.8)	7.9 (2.8)/6.9 (2.6)

**PANSS Exc**	**8.2 (3.8)/5.0 (1.9)** **∗**	**9.3 (5.4)/5.5 (2.4)** **∗** **∗**

**PANSS Dep**	6.8 (3.7)/5.8 (2.6)	6.1 (3.4)/5.5 (2.9)

**IS severity**	**1.9 (2.3)/0.5 (1.6)** **∗**	1.13 (2.1)/0.5 (1.8)

**IS intensity**	**6.8 (8.4)/1.2 (3.8)** **∗**	3.9 (7.1)/2.5 (5.5)

**RSQ**	**21.2 (6.1)/23.4 (6.6)** **∗**	22.4 (3.9)/23.6 (5.1)

**Social cognition**	80.6 (8.0)/83.5 (14.9)	81.9 (12.1)/84.3 (8.4)

*∗*=*p*<0.05; *∗∗*=*p*<0.001.

**Table 3:** Between group changes from baseline to end point in clinical and cognitive measurements.

**Change from baseline**	**Recent SZ (n=10)**	**Long-term SZ (n=15)**	***p* value**
	Mean (SD)	Mean (SD)	
**GAF **	12.830 (2.070)	9.642 (1.855)	**0.004**
**PANSS Pos**	3.154 (1.125)	4.705 (1.008)	0.101
**PANSS Neg**	2.748 (0.744)	1.377 (0.666)	**0.025**
**PANSS Dis/Con **	1.183 (0.509)	1.348 (0.456)	0.403
**PANSS Exc**	3.478 (0.643)	3.935 (0.576)	0.056
**PANSS Dep**	0.760 (0.648)	0.616 (0.580)	**0.022**
**IS severity**	4.837 (1.402)	2.303 (1.256)	**0.005**
**IS intensity**	4.837 (1.402)	2.303 (1.256)	**0.022**
**RSQ**	2.798 (0.614)	1.502 (0.550)	0.313
**Social cognition**	6.252 (2.450)	-0.325 (2.195)	0.061

## Data Availability

The data used to support the findings of this study are available from the corresponding author upon request.
